# Supplementary material for: Systemic vasoconstriction and mortality in patients with heart failure and reduced ejection fraction: A cohort of patients who underwent non-invasive hemodynamic monitoring
Source: PLoS One. 2024 Dec 20;19(12):e0312582. doi: 10.1371/journal.pone.0312582 (PMC11661613; doi:10.1371/journal.pone.0312582)
Supplement: Attachment — Submitted filename: RESPONSE TO REVIEWERS_JUL24.docx [file pone.0312582.s003.docx]

Plos One

PONE –d-24-10329

Deadline Jul 24 2024

To Yashedra Sethi

Academic Editor – Plos One

We thank you and the reviewers for your careful revision of our manuscript. We hope that our answers and corrections based on these suggestions meet your expectations and our work is now acceptable for publication in your esteemed journal.

Best regards

Marcelo Eidi Ochiai, on behalf of all authors.

Reviewer #1: “In this article, the authors analyzed the use of Medflow (non invasive hemodynamic monitoring) to evaluate the systemic vascular resistance and determine if it predicts mortality in patients with ADHF.

We have some doubts about methodology:

1. How the SVRi was calculated (there is no mention of de CVP in the formula used). In the article it is mentioned that the SVRi was calculated by dividing the mean arterial pressure by the cardiac index.”

The formulas are:

$$V_{Z} = A_{SYS}\div Z_{AO}$$

$$V_{CZ} =V_{Z} \left[ 0.66 +0.005 \times HR -0.01 \times age \times\left( 0.014 \times Pmean -0.8 \right) \right]$$

$${CO}_{CZ} =V_{CZ} \times HR \times cal$$

$$SVRI =Pmean \div\left( {CO}_{CZ}\div BSA \right)$$

Where V_Z_ is the stroke volume, A_sys_ is the area under the systolic portion of the arterial pressure wave, Z_ao_ is the aortic impedance, V_CZ_ is the corrected stroke volume, HR is the heart rate, Pmean is the mean arterial pressure, CO_CZ_ is the Wesseling's pulse contour cardiac output, the calibration factor is cal=CO_CZ_/CO_ref_, SVRI is the systemic vascular resistance index, and BSA is the body surface area.

(Lines 88 to 96)

2. “In the baseline there is no mention about the use of inotropic drugs and if the acquisition of RVRi was priorily the introduction of inotropic.”

Forty-six patients (79%) received dobutamine during hemodynamic monitoring, as highlighted in the line 132.

3. “We think it is more appropriated to show the results in the final table (table2) divided in the two groups priorly analyzed (RVP <1200 and >1200). In the article the final analyses it was divided by outcome.”

Table 2. Mortality according to systemic vascular resistance indexed

| Variables | Higher SVRI  (n = 35) | Lower SVRI  (n = 23) | Odds ratio (95% CI) | *P-value* |
| --- | --- | --- | --- | --- |
| One-year mortality | 22 (64.7%) | 10 (43.5%) | 2.38 (0.81-7.04) | 0.143 |
| Three-year mortality | 29 (85.3%) | 14 (60.9%) | 3.88 (1.08-13.89) | 0.037 |
| Total mortality | 31 (91.2%) | 20 (87.0%) | 1.55 (0.28-8.45) | 0.175 |

SVRI = systemic vascular resistance index; higher SVRI: >1,200 dyn∙s∙cm^−5^∙m^−2^; lower SVRI <1,200 dyn∙s∙cm^−5^∙m^−2^.

Reviewer #2: “I had the pleasure of reviewing the manuscript titled "Systemic Vasoconstriction and Mortality in Patients with Heart Failure and Reduced Ejection Fraction: A Cohort of Patients Who Underwent Non-Invasive Hemodynamic Monitoring." The manuscript effectively discusses and evaluates the prognostic significance of non-invasively identified systemic vasoconstriction in patients with decompensated heart failure and reduced ejection fraction (HFrEF). It should be free of grammatical and typographical errors. For instance, on line 141, the word "according" is missing a "to" after it. I recommend that the manuscript be reviewed for similar mistakes”.

We thank the reviewer for carefully reading and thereafter correcting the grammatical errors made in our manuscript.
