# Supplementary material for: Systemic vasoconstriction and mortality in patients with heart failure and reduced ejection fraction: A cohort of patients who underwent non-invasive hemodynamic monitoring
Source: PLoS One. 2024 Dec 20;19(12):e0312582. doi: 10.1371/journal.pone.0312582 (PMC11661613; doi:10.1371/journal.pone.0312582)
Supplement: Attachment — Submitted filename: Response_to_Reviewer_3_SEPT_24.docx [file pone.0312582.s004.docx]

Answers

Reviewer #3: The authors have assessed the utility of a non-invasive hemodynamic method to measure peripheral arterial resistance and to establish its relationship with mortality. This is an interesting study that might draw the attention of readers. However I have some comments to improve the quality of the manuscript.

Thank you for carefully reviewing our manuscript and providing suggestions. We have provided our responses below. We hope that our answers and corrections based on these suggestions meet your expectations.

1. Please add reference, page 3, lines 51 and 52. Mortality reduction with ACEi and hydralazine/nitrates.

We have added a reference at the relevant location.

2. Did the authors exclude patients with acute coronary syndromes as the cause of HF decompensation? Were there any cases of acute myocardial infarction?

Since the study was conducted in a heart failure unit, patients with angina, acute ischemic electrocardiographic changes and increased cardiac enzymes were not included as they are treated in the coronary care unit of our institution. To be clear, we included acute coronary syndromes as an exclusion criterion (lines 72-73).

3. The authors should clarify the exact moment when the hemodynamic evaluation was done. As I understood, it was done at admission, after the patients received the initial treatment and became stable. Is that correct? All patients underwent the hemodynamic evaluation within 24 h of admission?

We evaluated decompensation of heart failure after initial stabilization when the patient had minimal effort or no intravenous inotrope dependence. The hemodynamic monitoring was done 19 days (median) after admission with IQR of 28 days. In fact, all patients underwent the hemodynamic monitoring after 24 h of admission (line 78).

4. Statistics: It is not clear whether the authors did multivariate analysis. Was this multivariate logistic regression? Why did the authors use logistic regression? Since they constructed survival curves, I assume they have the date in which the events occurred. I believe it would be more appropriate to use as an endpoint the time to the event of mortality and use Cox proportional hazard models (univariate and multivariate analysis) to assess whether vasoconstriction was independently associated with mortality. This is a small sample but the number of events is elevated, allowing for multivariate analysis.

We used the logistic regression as multivariate analysis. Initially, we used Cox regression; however, we did not find difference between the groups’ higher and lower systemic vascular resistance. We attribute that finding to our long follow-up period, as most patients died, and hence, collapsing curves and a type II error was generated. The survival curves have shown a visual difference between the groups with more intensity at middle of the follow-up period. Therefore, we decided to analyze mortality at 1-year, 2-years, and 3-years post initial admission with logistic regression, which demonstrated a statistical significance (P=0.037) between the groups.

5. I understood that this is a retrospective study since patients were included back in 2009. If so, please add this information in methods, line 68.

The sample population of this study was assembled at beginning of the research protocol and we followed the patients all these years; therefore, ours is a prospective study (Fletcher and Fletcher, “Clinical Epidemiology: the essentials” chapter 5).

6. In table 3, we see that patients who died had higher LVEF% (24.1 vs 19.1%). This was unexpected. Did the authors find an explanation for that? Perhaps it was by chance?

We believe that probably this finding was by chance because of the small number of living patients at the end of follow-up period, although this was not statistical significant after multivariate logistic regression.

7. Please add p value in figure 2.

To provide more information, we have added *P-value* by log-rank test of total follow-up and *P-value* of 3-year mortality by logistic regression.

8. Please add in the limitations that this is a small sample.

Thank you for the valuable suggestion. We have mentioned small sample size as a limitation of the study (line 226).

9. Please comment in the discussion that in the ESCAPE trial, adjusting medications by hemodynamic profiles in patients with advanced HF did not reduce events as compared with clinical assessment alone. In addition, hemodynamics have been outperformed by neuro-hormonal assessment. So it is important to show in the manuscript that vasoconstriction was a predictor of mortality regardless of BNP.

We have included this aspect of invasive hemodynamic monitoring in the discussion. The changes are in lines 150, 153, 170, 171.
