# Supplementary material for: Systemic vasoconstriction and mortality in patients with heart failure and reduced ejection fraction: A cohort of patients who underwent non-invasive hemodynamic monitoring
Source: PLoS One. 2024 Dec 20;19(12):e0312582. doi: 10.1371/journal.pone.0312582 (PMC11661613; doi:10.1371/journal.pone.0312582)
Supplement: Attachment — Submitted filename: Reviewer Comments PLOS.pdf [file pone.0312582.s002.pdf]

Reviewer Comments

I had the pleasure of reviewing the manuscript titled "Systemic Vasoconstriction and Mortality in Patients with Heart Failure and Reduced Ejection Fraction: A Cohort of Patients Who Underwent Non-Invasive Hemodynamic Monitoring." The manuscript effectively discusses and evaluates the prognostic significance of non-invasively identified systemic vasoconstriction in patients with decompensated heart failure and reduced ejection fraction (HFrEF). It should be free of grammatical and typographical errors. For instance, on line 141, the word "according" is missing a "to" after it. I recommend that the manuscript be reviewed for similar mistakes.
